# Rifampin combination therapy in staphylococcal prosthetic joint infections: a randomized controlled trial

**DOI:** 10.1186/s13018-020-01877-2

**Published:** 2020-08-28

**Authors:** Øystein Espeland Karlsen, Pål Borgen, Bjørn Bragnes, Wender Figved, Bjarne Grøgaard, Jonas Rydinge, Lars Sandberg, Finnur Snorrason, Helge Wangen, Eivind Witsøe, Marianne Westberg

**Affiliations:** 1 Division of Orthopaedic Surgery, Oslo University Hospital, Oslo, Norway; 2 Department of Orthopaedic Surgery, Betanien Hospital, Skien, Norway; 3 Department of Orthopaedic Surgery, Martina Hansen Hospital, Bærum, Norway; 4 Department of Orthopaedic Surgery, Vestre Viken HF, Drammen, Norway; 5 Department of Orthopaedic Surgery, Bærum Hospital, Bærum, Norway; 6 Department of Orthopaedic Surgery, Sykehuset Innlandet HF, Lillehammer, Norway; 7 Department of Orthopaedic Surgery, Sykehuset Innlandet HF, Elverum, Norway; 8 Department of Orthopaedic Surgery, St. Olavs Hospital, Trondheim, Norway

**Keywords:** Rifampin, Staphylococci, Prosthetic joint infection, Surgery

## Abstract

**Background:**

The evidence supporting rifampin combination therapy in prosthetic joint infections (PJI) is limited due to the lack of controlled studies. The aim of this study is to evaluate the effect of adding rifampin to conventional antimicrobial therapy in early staphylococcal PJIs treated with debridement and retention of the implant (DAIR).

**Methods:**

In this multicenter randomized controlled trial, 99 patients with PJI after hip and knee arthroplasties were enrolled. They were randomly assigned to receive rifampin or not in addition to standard antimicrobial treatment with cloxacillin or vancomycin in case of methicillin resistance. The primary endpoint was no signs of infection after 2 years of follow-up.

**Results:**

Forty-eight patients were included in the final analyses. There were no differences in patient characteristics or comorbidities between the two groups. There was no significant difference in remission rate between the rifampin combination group (17 of 23 (74%)) and the monotherapy group (18 of 25 (72%), relative risk 1.03, 95% confidence interval 0.73 to 1.45, *p* = 0.88).

**Conclusion:**

This trial has not proven a statistically significant advantage by adding rifampin to standard antibiotic treatment in acute staphylococcal PJIs.

**Trial registration:**

The Regional Ethics Committee and the Norwegian Medicines Agency approved the study (EudraCT 2005-005494-29), and the study was registered at ClinicalTrials.gov at Jan 18, 2007 (NCT00423982).

## Highlights/summary

Rifampin is increasingly used in staphylococcal prosthetic joint infections treated with debridement and retention of the prosthesis. This study is the largest randomized controlled study on this subject. No statistical significant advantage by adding rifampin to the antimicrobial medication is shown.

## Introduction

The number of patients requiring prosthetic joint replacement is increasing due to good functional outcome and excellent pain relief in a growing population of the elderly [1]. Prosthetic joint infection (PJI) is a rare but devastating complication occurring in 1–2% of primary interventions [2, 3] and in 2–20% of revision procedures [4]. PJI leads to increased morbidity, long periods of hospitalization, and high costs [3, 5–7]. Of concern, the absolute number of PJI is increasing due to the increasing number of joint replacements. Also, the risk of infection seems to be increasing in recent years [8, 9].

Coagulase-negative staphylococci (CoNS) and *Staphylococcus aureus* are the most frequent cause of PJI, accounting for 30–47% and 12–44%, respectively [10–14]. Staphylococci are biofilm-forming bacteria [15]. The microbes adhere to prostheses and adjacent tissues and are enclosed in a polymeric matrix, where they are protected from the host immune response and antimicrobials. This makes the eradication of PJI difficult [16–18].

In acute PJI and acute hematogenous spread PJI, debridement and implant retention (DAIR) combined with antimicrobial treatment is an attractive surgical option due to its lesser surgical trauma and hence limited functional impairment, but the results vary greatly in the literature [13, 19, 20]. Rifampin is a broad-spectrum antimicrobial agent that is a frontline drug in the treatment of tuberculosis, but also acts bactericidal against *S. aureus.* It penetrates the biofilm and is able to kill sessile bacteria [21]. Due to the rapid development of resistance, rifampin must never be used alone, but in combination with another antimicrobial agent. Further, there are challenges with drug interactions, reported in up to 52% of patients treated with rifampin-combination therapy for infective endocarditis [22].

Rifampin appears to be promising in treating serious gram-positive implant-related infections [16, 23–25]. However, evidence to support the adjunctive use of rifampin in PJI-treatment is week and based on one small, randomized controlled trial and some observational studies [23–28]. Due to the absence of randomized controlled trials, and the limitations of the existing literature, there is still a debate regarding the role of rifampin in staphylococcal PJIs.

The aim of this trial was to evaluate the effect of adding rifampin to a standard antimicrobial therapy with cloxacillin or vancomycin in early postoperative and acute hematogenous staphylococcal PJI.

## Patients and methods

### Study design and participants

This open-label, randomized controlled trial was conducted in five district general hospitals, one specialized orthopedic hospital and two university hospitals in Norway. Recruitment was from January 2006 to January 2012, with a final follow-up scheduled at 2 years. Eligible patients were adult men and women operated with a total hip or knee prosthesis, with clinical signs suggesting early postoperative or acute hematogenous PJI and with a stable implant in place. Confirmed infections due to *S. aureus* or CoNS were included in the study. Positive cultures in the expected aseptic revision were not included. Exclusion criteria were PJI with other bacteria than staphylococci, less than 2 years expected survival, inability to comply with treatment and/or follow-up visits, and contraindications to the use of rifampin, cloxacillin, or vancomycin. The Regional Ethics Committee and the Norwegian Medicines Agency approved the study (EudraCT 2005-005494-29), and the study was registered at ClinicalTrials.gov (NCT00423982). Written informed consent was obtained from all patients before inclusion. The study has been performed according to the principles of the Declaration of Helsinki.

### Definition of PJI

PJI was suspected when patients presented with pain, redness, or wound discharge within 30 days after prosthetic surgery (acute postoperative PJI) or with an acute hematogenous PJI with symptoms for less than 3 weeks [13]. During the DAIR procedure, eight intraoperative tissue specimens were collected with separate instruments, of which one from periprosthetic bone and one from synovial fluid. At least two of the specimens had to be positive with the same microbe to define PJI.

### Randomization

Patients were randomized at admission to hospital to conventional antimicrobial therapy with or without the adjunction of rifampin. Randomization was stratified by center and performed by a randomization generator by blocks of 10.

### Surgical treatment

All included patients underwent a highly standardized soft tissue revision, with thorough debridement including excision of the wound. New instruments were introduced after suprafascial incision. The implants were left in place, but modular components were exchanged. The wound was cleansed with pulsatile irrigation with 9 L of saline. After the DAIR procedure, new draping and instruments were introduced, including the new modular components. Two 10 × 10 cm gentamicin-containing collagen sponges, each containing 130 mg gentamicin sulphate, were placed in the wound before closure. Finally, the wound was sutured in layers. No drains were used.

### Antimicrobial therapy

The first dose of antibiotics was given perioperatively immediately after the 8 tissue specimens were collected. All patients were given cloxacillin 2 g × 4 and vancomycin 1 g × 2 intravenously until microbiological results were known. Patients randomized to the rifampin-combination group were in addition treated with oral rifampin from day 1 after surgery. When cultures proved methicillin-susceptible *S. aureus* or CoNS, rifampin 300 mg × 3 orally and cloxacillin 2 g × 4 intravenously were given for 2 weeks, then rifampin 300 mg × 3 orally and cloxacillin 1 g × 4 orally for 4 weeks. In case of methicillin-resistant *Staphylococcus epidermidis* (MRSE), patients were treated with rifampin 300 mg × 3 orally and vancomycin 1 g × 2 intravenously for 6 weeks. In the monotherapy group, when proven methicillin-susceptible staphylococci, cloxacillin 2 g × 4 intravenously was given for 2 weeks, then cloxacillin 1 g × 4 orally for 4 weeks. In case of MRSE, patients were treated with vancomycin 1 g × 2 intravenously for 6 weeks.

Vancomycin serum levels were monitored 2 times per week, and the vancomycin dose was adjusted if the serum level outranged the recommended plasma concentration levels.

### Follow-up

Medical conditions and medications prior to surgery were recorded, as well as demographic data. Patients were clinically assessed at enrolment, during the hospital stay, and regularly throughout the treatment period. Hematological status, serum-creatinine, and hepatic enzymes were analyzed before treatment and during antimicrobial treatment to determine any toxic side effects. C-reactive protein (CRP) and erythrocyte sedimentation rate (ESR) were used to assess treatment effectiveness.

The cure was defined as the lack of clinical signs and symptoms of PJI (fever, joint pain, erythema, warmth of the skin around the incision, and sinus tract), CRP < 10 mg/ml, ESR as prior to index operation, and no radiological signs of loosening at 2 years of follow-up.

Confirmed failure was defined as re-revision with the isolation of the initial or other microorganisms from a minimum of two intraoperative tissue specimens during the 2-year study period. Probably, failure was defined if clinical signs and symptoms of local infection but without microbiological documentation. Both groups were considered failures in the analysis. Repeated DAIR procedures were considered being a failure.

### Statistical analysis

Based on data from in-hospital quality registers, we assumed a cure rate of 70% following a DAIR procedure without the addition of rifampin. An increase in the cure rate of 20% may be proven with a statistical power of 80% when including 62 patients in each group. Taking into account the expected dropouts, we intended to include 100 patients in each group.

Analyses were conducted according to a modified intention-to-treat principle. Time to failure was estimated with the Kaplan-Meier method, and the log-rank test was used to compare groups. The chi-square or Fisher’s exact test was used to compare categorical and continuous variables in the rifampin group and the monotherapy group. A *P* value < 0.05 was considered significant. We used SPSS for Windows, version 23 (SPSS inc, Chicago, IL, USA) for the analyses.

## Results

**Fig. 1:**
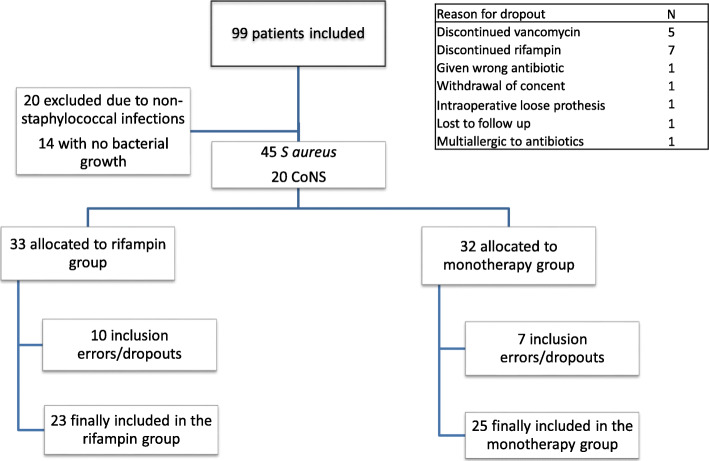
Flowchart

Overall, 99 patients with suspected PJI were enrolled in the study, of which 65 had a proven staphylococcal infection. The last follow-up visit was in January 2014. Recruitment for the trial was slower than anticipated, and the study was stopped before reaching the estimated sample size. In addition, an increasing trend towards using rifampin developed in the orthopedic society during the study period, which also made inclusion more difficult. Figure 1 displays the study profile.

Forty-eight patients were included in the final analyses, 23 in the rifampin-combination group, and 25 in the monotherapy group. Baseline characteristics were similar between the groups (Table 1). The median age was 68.5 years (range 37–92). *S. aureus* was found in 36 of the participants and CoNS in 14. Two patients had a combination of *S. aureus* and methicillin-susceptible *S. epidermidis*. Further, there was one infection with *Staphylococcus lugdunensis* and one *Staphylococcus capitis*, both susceptible to methicillin (Table 2).

**Table 1:** Baseline characteristics of the 48 patients

Characteristics	Rifampin group (*n* = 23)	Monotherapy group (*n* = 25)	Total (*n* = 48)
Age, year, median (range)	70 (37–92)	66 (39–84)	68.5 (37–92)
Sex, male (%)	15 (65)	17 (68)	32 (67)
ASA scores 1–2, no (%)	16 (70)	21 (84)	37 (77)
BMI, mean (SD)	30.1 (1.3)	27 (1.0)	28.4 (0.8)
Diabetes mellitus	3	3	6
Immunosuppressive medication	2	2	4
Smoking	3	4	7
Time from index surgery to revision, median, days (range)	19 (7–912)	17 (8–122)	18 (7–912)
Hip prosthesis			
Primary hip prosthesis	17	14	31
Revision hip prostehesis	3	5	8
Knee prosthesis			
Primary knee prosthesis	3	6	9
CRP pre surgery, mean (SD)	135 (21.1)	167 (26.4)	151 (16.9)
Creatinin pre surgery, mean (SD)	78 (5.7)	79 (4.4)	79 (3.5)
Type of prosthesis^a^			
Cemented prosthesis	14	16	30
Non cemented	4	5	9
Reverse hybrid	4	4	8

*ASA* American Society of Anesthesiologists physical status classifications system, *BMI* body mass index

^a^Missing data, *n* = 1

**Table 2:** Bacterial findings in initial DAIR procedure

Microbes	Rifampin-combination group	Monotherapy group	Total
MSSA	15	19	34
MRSE	5	5	10
MSSA + MSSE	2	0	2
*Staph lugdunensis*	1	0	1
*Staph capitis*	0	1	1

*MSSA* methicillin-susceptible *S. aureus*, *MRSE* methicillin-resistant *S. epidermidis*

The median follow-up was 27 months (range 18–99) in the rifampin-combination group and 27 months (range 7–106) in the monotherapy group. Some of the latest follow-ups were conducted as telephone interviews. The two patients with the shortest follow-up were deceased before final the follow-up, but were reported to be infection-free at the time of death.

### Outcome at 2 years

**Fig. 2:**
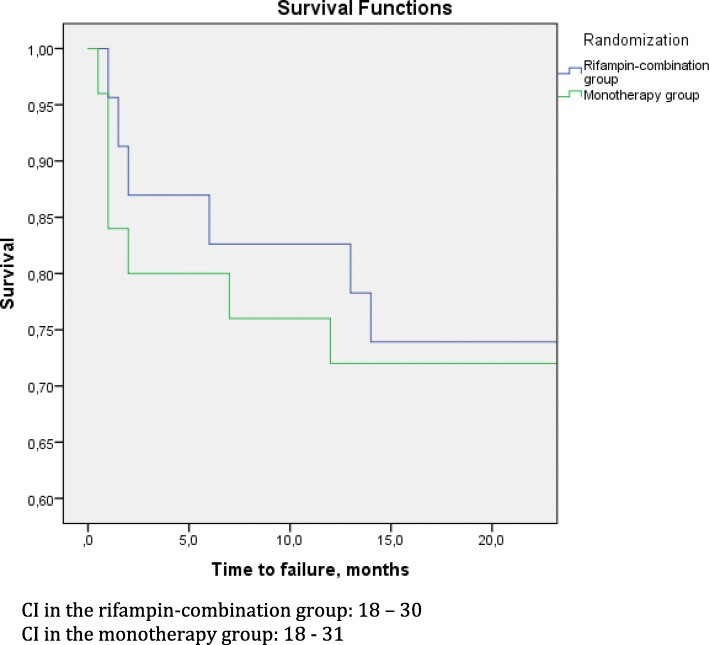
Kaplan Meier survival curve

There was no statistically significant difference between the two treatment groups in the success of DAIR procedure in the eradication of acute staphylococcal PJI; the success rate at 2 years was 17/23 (74%) in the rifampin-combination group and 18/25 (72%) in the monotherapy group (95% CI 0.73–1.45; *p* = 0.88). A successful DAIR procedure is of important clinical relevance for the patients, as further revision arthroplasty, and thereby possible functional impairment, is not needed. A Kaplan-Meier plot is used to show time to failure in the two groups (Fig. 2).

Subgroup analyses according to the type of staphylococci showed a cure rate for *S. aureus* infections of 14/18 in the rifampin group and 13/20 in the monotherapy group (95% CI 0.80–1,80; *p* = 0.49). CoNS infections had a cure rate of 5/5 in the monotherapy group and 3/5 in the rifampin-combination group (95% CI 0.29–1.22; *p* = 0.44) (Table 3). These two groups were also similar in age, sex, and comorbidities.

**Table 3:** Subanalyses according to type of staphylococci

^a^		Success	Failure
Rifampicin group	MSSA	14/18	4/18
	MRSE	3/5	2/5
Monotherapy group	MSSA	13/20	7/20
	MRSE	5/5	0/5
Total	MSSA	27/38	11/38
	MRSE	8/10	2/10

^a^MSSA methicillin-susceptible *S. aureus*, *MRSE* methicillin-resistant *S. epidermidis*

The bacteria found in the revisions after failures are listed in Table 4. There was no development of rifampin resistance in the two patients from the rifampin-combination group with positive cultures.

**Table 4:** Bacterial findings at rerevisions due to failure

Microbes	Rifampin group	Monotherapy group
MSSA	0	2
MRSE	2	1
MRSE + *Enterococcus faecalis*	0	1
No growth	2	3
Not taken	1	0
Not revised	1	0
Total	6	7

### Adverse events

Of the 65 patients initially included in the study, only 4 of 31 who were assigned to rifampin treatment dropped out or were excluded due to side effects or discontinuation of rifampin. One patient using rifampin was reported with hepatic failure. For vancomycin, 5 patients dropped because of side effects, but as many as 13 patients developed increased serum-creatinine levels that led to the discontinuation of vancomycin. Eight out of these 13 continued in the study because their bacteria resistance pattern allowed them to continue treatment without vancomycin. The causes of inclusion errors or drop-outs are listed in Fig. 1, and the outcomes of the drop-out patients are listed in Table 5.

**Table 5:** Outcome of dropout patients*

Initial therapy	Therapy after dropout	Cure	Failure
Cloxacillin and rifampin	Cloxacillin	4	1
	Cloxacillin and cotrimoxazol	1	
Vancomycin and rifampin	Vancomycin and linezolid	1	
	Rifampin and linezolid	1	
Cloxacillin	Cloxacillin	1	
	Fusidic acid and rifampin		1
	Ciprofloxacin and cloxacillin	1	
	Clindamycin	1	
Vancomycin	Linezolid and trimetoprim-sulfa		1
	Ciprofloxacin and cloxacillin	1	
	Clindamycin		1

*Two dropout-patients are not registred in the table due to withdrawal of consent and lost to follow-up

## Discussion

In this multicenter, randomized controlled trial involving 48 patients with acute staphylococcal PJI treated with a DAIR procedure, the addition of rifampin to standard treatment with cloxacillin or vancomycin did not improve the cure rate. To our knowledge, this is the second randomized controlled trial to examine the effectiveness of adjuvant rifampin therapy in acute PJIs, and our findings are in contrast to previous findings. Zimmerli et al. published the first study back in 1998 [23]. It was a single-center trial involving 24 patients, of which 15 were PJIs and 9 were infected osteosyntheses. This study was prematurely discontinued because all the failures occurred in the control group. It has been criticized for small numbers and limited statistical power, 33% drop-out rate in the rifampin group, as well as the choice of ciprofloxacin as monotherapy in the control group. Due to the concerns by using ciprofloxacin in monotherapy, we chose to add rifampin to the standard treatment at the time, which was cloxacillin or vancomycin in case of methicillin resistance.

Several retrospective observational studies and case series have been published in the last decades, evaluating different rifampin combinations. These studies were not controlled studies, and the success rates have never reached 100% as in the Zimmerli study, but their findings have although favored the use of rifampin [25, 27, 28]. There are difficulties in the interpretation of these studies, including considerable differences in baseline characteristics between treatment groups, surgical methods not described in detail, and varying MRSA rates. Barberán et al. found a success rate of 65% in staphylococcal PJIs treated with rifampin and levofloxacin following DAIR, seemingly a more common and expected result from these infections [29]. In a recently published study, a significant higher failure rate was found in rifampin combinations with linezolid, co-trimaxazole, and clindamycin. This is explained by the fact that rifampin reduces the serum concentration of these drugs and also for fusidic acid [30, 31]. Two review articles from 2008 and 2010, respectively, both conclude that the use of rifampin is based mostly on noncomparable in vitro and in vivo data and retrospective case reviews. Because of the biases of these, there are not sufficient data to support rifampin combination therapies [32, 33]. However, it suggests that it could be effective in infections containing biofilm-producing agents, such as staphylococcal PJIs, but its use must be evaluated against the probability of drug interference and toxicity for each individual patient. A retrospective study from 2017 proved no advantage in treating streptococcal infections with an addition of rifampin [34]. These infections also form biofilm, and rifampin should theoretically improve the outcome. In vitro studies are inconsistent, but most have shown that the combination of vancomycin and rifampin promotes antagonism or indifference [35]. An in vivo case report showed a higher failure rate in PJIs when combining vancomycin and rifampin after debridement and retention of the prosthesis [29]. This is consistent with our results and the combination vancomycin-rifampin should be used cautiously. An RCT on staphylococcal bacteraemia proved no benefit from adding rifampin to the standard antibiotic therapy [36].

The preferred treatment in early PJI is DAIR. This option reduces morbidity, improves function, and is cost-effective compared to 1- and 2-stage revisions [11, 37]. It has been postulated that the biofilm has increased to such a degree that cure with DAIR is less achievable after 1 month [38]. Only patients with infections within 4 weeks after surgery, and acute hematogenous infections, were included in our study. The reported results following DAIR have been varying considerably, ranging from 21% to 100% [13, 19, 23, 27, 39]. There are many limitations when interpreting the literature, as several factors are varying and the surgical procedures are often poorly described. Both the definition of acute PJI, the number of procedures, the type and duration of antibiotics, and even the definition of success vary. Our results of approximately 75% success at 2 years of follow-up without suppressive antimicrobial therapy are comparable with recent literature [40, 41]. Regarding the number of procedures, some advocates that repeated DAIR are effective [42]. On the other hand, some authors have found the need for additional DAIRs is associated with increased risk of failures [43]. In this present study, we chose to regard an additional DAIR procedure as a failure.

We found *S. aureus* to be more frequent than CoNS in this material, which is in contrast to most reports [12, 14, 30]. This may reflect the challenges of defining PJI. We used the Tsukayama definition, including patients with a short duration of symptoms shortly after the index surgery (30 days) [13]. Many definitions include PJI within the first 3 months after index surgery as early postoperative infections. This may explain the findings of more virulent bacteria (*S. aureus*) in our material.

PJI caused by methicillin-resistant *S. aureus* (MRSA) is reported to worsen the prognosis of DAIR, and a rifampin-containing antimicrobial regimen is often used. There were no MRSA infections in our cohort, reflecting the still very low prevalence of these infections in the Nordic countries. A prevelance < 1% is reported in Norway over the last 20 years [44].

The strengths of the trial are its randomized, multicenter design, which provides generalizable findings. It is also the largest RCT to date examining adjunctive rifampin in the treatment of staphylococcal PJI. There are several limitations to the study. First of all, the sample size is relatively small, but the 65 patients included doubles the numbers included in the previous trial. Recruitment rates were lower than expected at all participating sites, illustrating why conducting randomized clinical trials in clinical settings with few eligible patients is difficult. The study was stopped after enrolment of 99 patients, without knowledge of study outcome. A small sample size increases the risk of type II error and then often showing high intervention effects. In this study, there was an independent reason for stopping the trial, which may lower the risk of bias. One may argue that statistical power is a pre-study tool, and because our study ended due to slow inclusion rates, the actual results may be interpreted as they are, based on the 95% CIs [45]. The 95% CIs around our estimates of the difference between the groups were quite small. Secondly, gentamicin collagen sponges were placed in the wound before closure. This adds an additional antimicrobial agent to the equation and was performed according to the guidelines at the time of initiating the study. However, all patients received these sponges, and it is difficult to see how it could have affected the final outcome. Third, the type of antibiotics combined with rifampin seems to affect the outcome. Our results may therefore not be comparable with other rifampin combinations.

In conclusion, our findings indicate that adding rifampin to cloxacillin or vancomycin treatment in patients with acute staphylococcal PJIs does not affect the cure rate of a DAIR procedure. Given the substantial risk of drug interactions and the risk of increasing bacterial resistance, we find that adjunctive rifampin provides no benefit over standard treatment. The results must however be cautiously interpreted due to a low number of patients, but the study still adds important knowledge to defining the benefit of rifampin.

## Data Availability

The datasets used and/or analyzed during the current study are available from the corresponding author on reasonable request.

## Abbreviations

PJI: Prosthetic joint infection
DAIR: Debridement, antibiotics, and implant retention
MRSE: Methicillin-resistant *Staphylococcus epidermidis*
CoNS: Coagulase-negative Staphylococci
CRP: C-reactive protein
ESR: Erythrocyte sedimentation rate
MRSA: Methicillin-resistant *Staphylococcus aureus*
MSSA: Methicillin-susceptible *Staphylococcus aureus*
CI: Confidence interval
